# Solution‐Processed Multi‐Junction Optoelectronic Synapses for Mode‐Switchable SWIR Neuromorphic Vision Sensing

**DOI:** 10.1002/advs.77617

**Published:** 2026-09-27

**Authors:** Zhuoyang He, Dae Yang Oh, Yi Ji, Yanyan Li, Chengwei Shan, Soong Ju Oh, Tianshuo Zhao

**Affiliations:** ^1^ Department of Electrical and Computer Engineering The University of Hong Kong Hong Kong SAR China; ^2^ Department of Materials Science and Engineering Korea University Seoul Republic of Korea; ^3^ Materials Innovation Institute for Life Sciences and Energy (MILES) HKU‐SIRI Shenzhen China

**Keywords:** dual‐mode, photodetector, photosynapse, quantum dot, shortwave infrared

## Abstract

Shortwave infrared (SWIR) optoelectronics are the main pillars for machine vision and perception. However, conventional photodetectors and neuromorphic sensors with complementary characteristics are more challenging to integrate into a single device architecture in the SWIR band. Herein, a bias‐programmable back‐to‐back rectifying junction is designed to switch between high‐speed photodetector and persistent optoelectronic synapse modes. Numerical models demonstrate how bias tunes the relative barrier heights of the multi‐junction stack, thereby modulating the carrier dynamics and triggering transitions among different operation modes. This concept is experimentally implemented using IR‐absorbing colloidal lead sulfide (PbS) quantum dots (QDs) assembled into anti‐series p‐n and Schottky junctions. For 1550 nm operation, cascaded p‐n QD homojunctions effectively improve photodetector performance while strengthening the synaptic plasticity of photocurrent. The programmable device states enable image denoising and motion trajectory tracking. Notably, a unique synapse regime is identified to suppress static IR background and enhance the signal‐to‐noise ratio (SNR) of dynamic events, achieving static‐dynamic fusion in SWIR vision. This solution‐processable and voltage‐controlled multi‐junction design provides a scalable route toward spatiotemporal SWIR vision sensing systems.

## Graphical abstract

A novel bias‐programmable shortwave infrared (SWIR) vision sensor is developed using multi‐junction colloidal quantum dots (QDs). Under varying applied voltage, the back‐to‐back rectifying junctions effectively tune trapping centers to switch the device between high‐speed photodetector and persistent synapse modes. This reconfigurable design enables advanced neuromorphic capabilities, including motion trajectory tracking and static background suppression, advancing intelligent spatiotemporal perception.

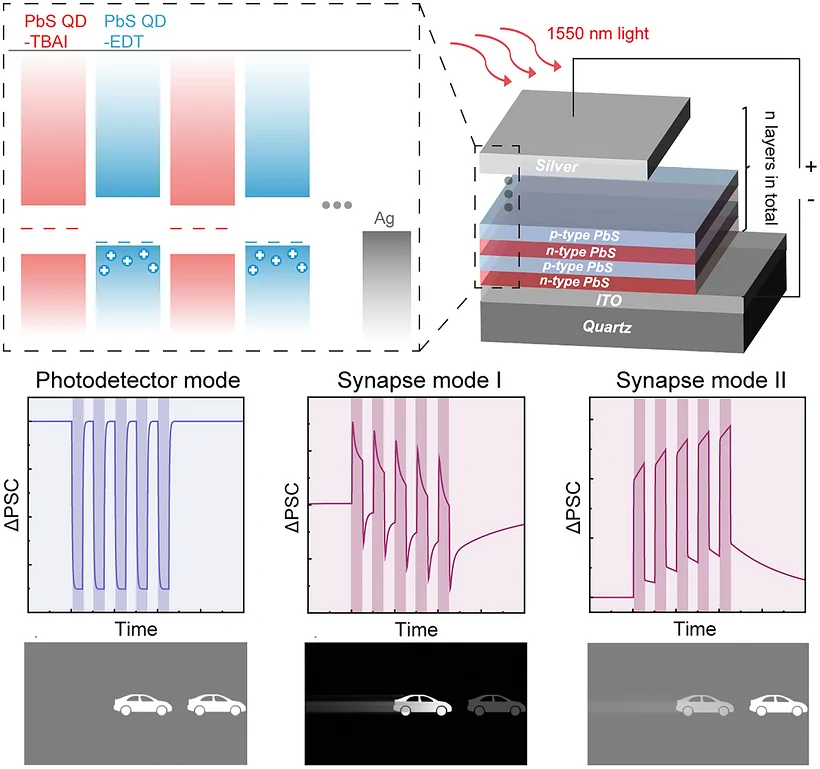

## 1 Introduction

Shortwave infrared (SWIR) photodetection is a crucial enabler for sensing, perception, and communication [1, 2, 3]. Conventional SWIR photodetectors typically rely on epitaxially grown InGaAs photodiodes to pursue high‐speed, high‐resolution imaging. However, this frame‐by‐frame collection mechanism can generate a large amount of redundant data, consuming computational and power resources. Neuromorphic devices have emerged as a promising solution to overcome the bottleneck of conventional Von Neumann architectures [4, 5, 6, 7]. One common type of neuromorphic device, namely, optoelectronic synapses, produces non‐linear history‐dependent photoresponse and mimics the fundamental weight‐tuning mechanisms of biological neural networks [8]. This synaptic plasticity enables these devices to implement advanced functionalities, such as enhancing transient signals from noise [9, 10], constructing artificial neural networks for deep learning [11, 12, 13], and extracting dynamic motion trajectories while suppressing static backgrounds [14, 15, 16]. Thus, such low‐power‐consumption neuromorphic device can supplement the traditional photodiode detectors to empower in‐sensor processing features and enhance SWIR imaging and sensing. Despite these advantages, a versatile device design that simultaneously supports both photodiode and synapse characteristics is still challenging, especially for the SWIR band with limited absorber material designs, leaving a blank for multi‐functional SWIR vision systems with efficient photodetection and in‐sensor neuromorphic perception.

Originating from their fundamentally distinct carrier dynamics, photodetector and synapse modes are built on instant carrier extraction and prolonged carrier storage, respectively. Accommodating their drastically different performance requires the device operation mechanism to be tunable and even switchable. Existing strategies to integrate photodetector and optoelectronic synapse modes within a single device include external gate voltage (V_g_) [17, 18, 19] and bias voltage (V_bias_) [20, 21, 22, 23] regulation and device configuration shifts [24]. More recently, a reconfigurable dual‐mode sensor architecture has been demonstrated to emulate the “dynamic‐static fusion vision” inspired by chameleon binocular systems [14]. The InGaN nanowire/hydrogel heterojunction configuration grants high‐performance photodetection and photosynaptic operation to identify both motion trajectories and absolute spatial positions in ball‐tracking tasks. However, most of these device schemes are not targeted at SWIR or longer wavelengths, where the involvement of narrow bandgap photoabsorbers, such as colloidal lead sulfide (PbS) quantum dots (QDs), limits built‐in potentials for driving charge carrier migration. More severe trade‐offs often occur between photodetector metrics (noise, responsivity, response time) and synaptic plasticity in the IR range. Therefore, specialized design strategies are required to precisely modulate SWIR photocarrier dynamics, concurrently enhancing the performance of both modes.

Here, we propose a solution‐processed multi‐junction optoelectronic device architecture based on colloidal PbS QDs that addresses three critical challenges in neuromorphic sensing: SWIR spectral operating at 1550 nm, bias‐programmable multi‐mode, and baseline‐suppression‐enabled dynamic vision sensor (DVS)‐like sensing. Through COMSOL electrostatic simulation, we observe the transient photocurrent generated by the multi‐junction stack under pulsed illumination. The simulated device characteristics are altered between a high‐speed photodetection state and a persistent synapse mode by varying the applied bias. We investigate the bias‐dependent carrier distribution step‐by‐step following a pulse duration and unveil detailed changes in the relative barrier heights of the back‐to‐back rectifying junctions. To experimentally verify this general strategy, we choose PbS QDs as the active IR‐absorbing materials for their solution‐based fabrication, size‐tunable bandgaps, and controllable doping concentrations [2, 25, 26]. Utilizing p‐type 1,2‐ethanedithiol (EDT) and n‐type tetrabutylammonium iodide (TBAI) ligand‐treated PbS QD films, we fabricate a multi‐layer device composed of indium tin oxide (ITO)/PbS QD‐TBAI/PbS QD‐EDT/silver (Ag), achieving a p‐n homojunction and a Schottky junction connected in anti‐series. The measured device characteristics are dual‐mode switchable, consistent with the simulation prediction. To further reduce the dark current and enhance responsivity to SWIR excitation, we leverage the size‐dependent QD bandgap and construct the junction with a near‐IR (NIR)‐absorbing PbS QD layer and a SWIR‐absorbing PbS QD layer to introduce higher potential barriers [27]. Furthermore, taking advantage of the facile layer‐by‐layer spin coating fabrication of QD films, we explore multiple p‐n homojunctions in cascade by alternating PbS QD‐EDT and PbS QD‐TBAI layers. The six‐layer cascade junction device shows the optimized performance at SWIR wavelength, suppressing the dark current density by 160 times in the photodetector mode and increasing the paired pulse facilitation (PPF) of the synapse mode by 13%. Finally, we simulate a SWIR neuromorphic vision system based on parameters of the switchable optoelectronic device arrays. Effective image denoising and dynamic trajectory tracking are achieved by the synapse mode, while instant switching between the photodetector and synapse modes enables vision fusion. We also address the challenge of static background noise in the synapse‐based imaging by precisely tuning the bias to minimize the steady‐state photocurrent, and hence, significantly enhancing the signal‐to‐noise ratio (SNR).

## 2 Results and Discussion

### 2.1 Design of Bias‐Dependent Optoelectronic Synapses Based on Back‐to‐Back Rectifying Junctions

**FIGURE 1:**
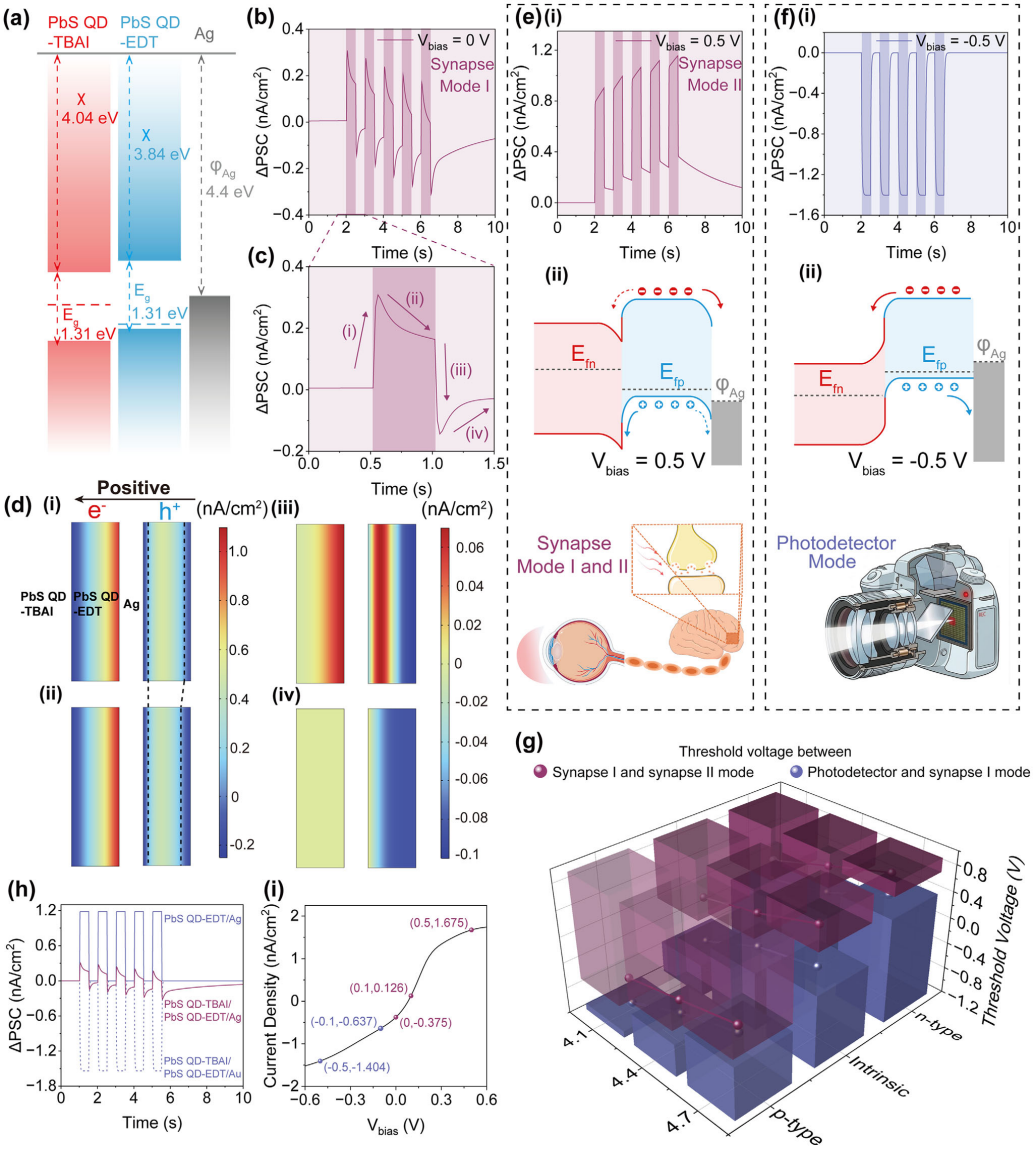
Design and simulation of the synaptic photoresponse of the back‐to‐back rectifying junction. (a) Band alignment of the PbS QD‐TBAI/PbS QD‐EDT/Ag structure before contact based on experimentally measured parameters. PSC of the device (b) under five consecutive pulses and (c) within a single pulse duration at zero bias (synapse mode I) (shaded regions indicate illumination‐on intervals). Labels (i) to (iv) represent four partitioned regimes. (d) Mapping of electron (left) and hole (right) current density distribution within the PbS QD‐EDT layer for the four regimes in (c), where current flow from right to left is defined as positive. Steps i and ii (during illumination) share the same color legend, where the black dashed line depicts the hole accumulation region. Steps iii and iv (recovery in the dark) share the same color legend. Comparison of PSC characteristics and corresponding band diagrams at (e) 0.5 V (synapse mode II) and (f) −0.5 V (photodetector mode) bias. Arrows represent the carrier transfer direction, with solid arrows representing the dominant direction. (g) Mapping of the threshold voltage defining the transition between photodetector mode and synapse mode I (magenta), and the transition between synapse mode I and synapse mode II (purple), as a function of metal work function and doping concentration of the PbS QD‐TBAI layer (p‐type: N_a_ = 1 × 10^15^ cm^−3^; Intrinsic: N_d_ = 2.7 × 10^8^ cm^−3^; n‐type: N_d_ = 1 × 10^15^ cm^−3^). (h) Comparison of simulated photocurrent characteristics among the back‐to‐back rectifying junction (magenta), PbS QD‐EDT/Ag (blue), and PbS QD‐TBAI/PbS QD‐EDT (purple) devices under zero bias. (i) Simulated steady‐state *I–V* characteristics of the back‐to‐back rectifying junction device under illumination.

We choose to design multiple semiconductor junctions within the same device stack for manipulating the carrier dynamics, which can be further regulated by tuning the barrier height of each junction through material engineering or external biasing. IR‐absorbing PbS QDs are selected as the model material system, as their films offer readily tunable Fermi levels through surface ligand treatment. Consistent with the literature [28], PbS QD thin films treated by solid‐state ligand exchange with TBAI (PbS QD‐TBAI) and EDT (PbS QD‐EDT) are weakly n‐type (N_d_ = 2.7 × 10^8^ cm^−3^) and strongly *p*‐type (N_a_ = 2.7 × 10^15^ cm^−3^), respectively, confirmed by valence band X‐ray and ultraviolet photoelectron spectroscopy (VB‐XPS and UPS) (Figure S1). The band diagram can be further derived by measuring their bandgaps from the Tauc plots of ultraviolet–visible (UV‐Vis) spectra (Figure S2). Therefore, as shown in Figure 1a, a p‐n homojunction is formed at the PbS QD‐TBAI/PbS QD‐EDT interface, while a Schottky junction is anticipated at the PbS QD‐EDT/Ag interface [29]. The entire layer stacking can construct two rectifying junctions in anti‐series, referred to as a back‐to‐back rectifying junction in the rest of this study. Based on this configuration, the energy band of the PbS QD‐EDT layer can bend downwards at both interfaces, creating a potential well for photogenerated holes in the valence band of the PbS QD‐EDT layer, while allowing photogenerated electrons to transport freely.

To quantitatively describe the energy landscape and study its trapping effects on transient photocurrent, we construct electrostatic simulations of such back‐to‐back junctions in COMSOL Multiphysics. Material trap densities are not included in the model, and an ohmic contact boundary condition is chosen for the PbS QD‐TBAI side, such that carrier trapping is exclusively investigated for the dependence of the multi‐junction band alignment [24, 30]. The simulated band diagram is shown in Figure S3, consistent with the back‐to‐back diode junction design. In the dark, the simulated *I–V* characteristics exhibit a bipolar rectifying behavior with different threshold voltages and rectification ratios for the two polarities (Figure S4), suggesting asymmetric barrier heights of the PbS QD‐TBAI/PbS QD‐EDT (*Φ_pn_*) and PbS QD‐EDT/Ag (*Φ_sc_*) junctions. For the equivalent circuit, we model the characteristics using a *p‐n* junction photodiode and a Schottky diode with different breakdown voltages connected back‐to‐back. When a sufficiently large positive or reverse bias is applied (**region I and III of** Figure S4), one of the diodes is turned on to show an exponentially growing current intensity [31].

The numerical model allows us to study the synaptic response of the device, i.e., the transient photocurrent upon illumination by periodic light pulses. Figure 1b shows the simulated photocurrent under zero bias in response to five consecutive light pulses with a 0.5 s duration at 808 nm wavelength, where the overall current level follows a unidirectional accumulation trend similar to the behavior of postsynaptic current (PSC) in other reported synaptic devices. However, the photoresponse within a single pulse period exhibits an atypical four‐regime behavior (synapse mode I) [32], as denoted by (i) to (iv) in the enlarged plot (Figure 1c). To illustrate the underlying mechanism, we map the current density distribution in the PbS QD‐EDT layer, which is the middle layer of the back‐to‐back rectifying junction. Upon illumination, the photocurrent magnitude rises rapidly, which is attributed to the net electron flux toward the Ag electrode (Figure 1d (i)). Herein, the current flow (the opposite direction as the electron current and the same direction as the hole current) from the Ag electrode to the opposing electrode is defined as positive. Meanwhile, the hole current is relatively low, contributing negligibly to the photocurrent in this regime. The simulated distribution suggests that holes are mainly confined within the PbS QD‐EDT layer, consistent with potential barriers formed at both junction interfaces based on the band bending simulation (Figure S3). This result also directly illustrates the hole‐trapping process by the back‐to‐back rectifying junction design at the beginning of the irradiation. As the illumination lasts steadily in regime (ii), the photocurrent amplitude relaxes back from the initial peak. Comparing the simulated current distributions in Figure 1d (ii) with (i), while the electron current component remains stable, the hole current density near the PbS QD‐EDT/Ag interface increases, indicated by the leftward shift of the boundary line (Figure 1d(ii), dashed). It is possibly due to the weakened* Φ_sc_* by increased local hole concentrations after phase (i), and such hole migration counters the electron current flow in the same direction, leading to a decrease in net photocurrent. Upon removing the light excitation, the electron current component and hence the overall photocurrent vanishes instantaneously (Figure 1d (iii)), whereas trapped holes in the QD layer continue to be released to the Ag contact through a delayed relaxation process, producing a transient negative photocurrent spike in regime (iii). In the final stage (iv), the negative photocurrent is dominated by the hole component, which decays in magnitude as holes detrap from the PbS QD‐EDT layer (Figure 1d (iv)). When the subsequent light pulse arrives before the end of the retention period, a second cycle of the above steps is triggered to occur with remaining carriers from the previous process, resulting in the synaptic behavior shown in Figure 1b.

To confirm that the synaptic behavior originates from the back‐to‐back rectifying junction, two control groups containing either one of the two single junctions are simulated. Replacing the Ag electrode with high work‐function Au, the Schottky contact is removed, and the PbS QD‐TBAI/PbS QD‐EDT homojunction alone leads to typical photodiode characteristics with linear photoresponse of the opposite polarity corresponding to the identical light pulses (Figure 1h). Similarly, when the PbS QD‐TBAI layer is removed, the single PbS QD‐EDT/Ag Schottky junction shows memoryless photocurrent, but the current direction is reversed to that of the PbS QD homojunction (Figure 1h). Although both single‐junction structures create built‐in electric fields to facilitate charge separation, the unique trapping phenomena and history‐dependent photocurrents are exclusively observed in the multi‐junction structure, proving the critical role of both junction interfaces in the photosynaptic behavior.

We find that this synaptic plasticity is highly tunable via V_bias_, which essentially tunes the relative potential barrier height of the two junctions through *Φ_pn/sc_^’^ =*  *Φ_pn/ sc_* − V_bias_. Figure 1e,f showcases the photoresponse and corresponding band diagrams under two opposite biases. When V_bias_ = 0.5 V is applied to the Ag electrode, the Schottky junction is reverse‐biased, while the *p‐n* homojunction is forward‐biased. Under illumination, the increased *Φ_sc_* further suppresses hole collection but enhances electron transfer at the Ag electrode (Figure 1e (ii)), while the decreased *Φ_pn_* favors the hole flux toward the PbS QD homojunction. Instead of migrating along the same direction in the zero‐bias case, here photogenerated electrons and holes transport toward the opposite electrodes and add up to form a growing positive photocurrent, resulting in step‐like potentiation of the PSC under successive optical pulses (synapse mode II), as shown in Figure 1e (i). The intrinsic band offset between the PbS QD‐TBAI and PbS QD‐EDT layer ΔE_v_ = 0.2 eV (Figure 1a) acts as a bias‐independent potential barrier to ensure hole trapping for the synapse operation mode under positive bias.

In contrast, a negative bias of −0.5 V on the Ag electrode can forwardly bias the Schottky junction, flattening its band bending induced by the junction interface, and concurrently reverse‐bias the PbS QD homojunction. In this regime, the device transforms into a photodiode dominated by the homojunction as shown in Figure 1f (ii), where photocarriers are separated and drifted efficiently along the potential gradient without significant trapping (Figure 1f (i)). As a result, the photocurrent rises and falls immediately following the change in illumination intensity, and the photocurrent direction becomes negative.

Compared to V_bias_ = 0.5 and −0.5 V, Figure S5 shows that intermediate biases of 0.1 and −0.1 V lead to non‐linear PSC features, belonging to the synapse mode I, as those of the non‐biased device. For these small bias magnitudes, the difference between *Φ_sc_* and *Φ_pn_* is slightly modified, and photogenerated electrons and holes still prefer to simultaneously transfer to the PbS QD‐EDT/Ag interface, undergoing the four processes shown in Figure 1c. However, the specific barrier heights of the back‐to‐back rectifying junctions determine the photocurrent change for each step, which is further modulated by the applied bias.

The correlation between the barrier height and bias‐dependent photoresponse mode is generalizable. We simulate the transient photocurrent while varying the barrier height at both junction interfaces. Specifically, the metal contact work function and the PbS QD‐TBAI layer Fermi level (doping concentration) are swept stepwise, while maintaining the PbS QD‐EDT layer parameters constant. As shown in Figure 1g, for a fixed work function and doping concentration, two threshold values exist for the bias voltage that distinguish three operational regimes: the photodetector mode (purple region), synapse mode II (magenta region), and synapse mode I (the transition region in between). When the work function of the metal contact is higher to yield a weaker *Φ_sc_* with the PbS QD‐EDT film, the threshold voltage required to lower *Φ_sc_* and turn on the Schottky diode is reduced, thereby facilitating the transition from the synapse mode I to photodetector mode at a lower negative bias (Figure 1g). On the other hand, a more significant *Φ_pn_*, formed by increasing the n‐type doping of the PbS QD‐TBAI layer (from nearly intrinsic N_d_ = 2.7 × 10^8^ cm^−3^ to N_d_ = 1 × 10^15^ cm^−3^), requires a larger positive bias to offset the built‐in potential and forward bias the p‐n diode. Further increasing the positive bias across this threshold voltage redirects the photogenerated holes in the PbS QD‐EDT layer toward the n‐type region, triggering the switch from synapse mode I to mode II (Figure 1g). However, when the PbS QD‐TBAI layer is set to be p‐type (N_a_ = 1 × 10^15^ cm^−3^), the barrier height and band bending at the QD homojunction are significantly decreased, leading to more sensitive dependence on the forward bias and smaller operation windows for the synapse mode I. The simulation results demonstrate that the mode‐switching threshold voltages and hence the photodetection mode are tunable by designing the barrier heights of both junction interfaces.

The accumulative synaptic effect can also be manifested by comparing the transient PSC with the steady‐state photocurrent under saturated illumination. Figure 1i shows the simulated steady‐state I‐V characteristics of the device with V_bias_ ranging from −0.6 to 0.6 V, which also represents the saturated photocurrent under continuous incidence. For an optoelectronic synapse, the transient photocurrent progressively approaches the steady‐state value with the number of optical pulses, such as the synaptic photocurrent shown in Figure 1e (i) becoming intensified to reach the current density obtained at V_bias_ = 0.5 V in Figure 1i. However, the output of the photodiode is insensitive to the duration or number of the light pulses, showing the same intensity in the transient (Figure 1f (i)) and steady‐state response (Figure 1i) under −0.5 V bias. This comparison further confirms the bias‐tunable switching between the two detection modes, namely history‐dependent synapses and instantaneous photodetectors, allowing dynamic‐static fusion perception, which will be demonstrated in the following sections.

### 2.2 Fabrication and Characterization of QD‐based Optoelectronic Synapses

**FIGURE 2:**
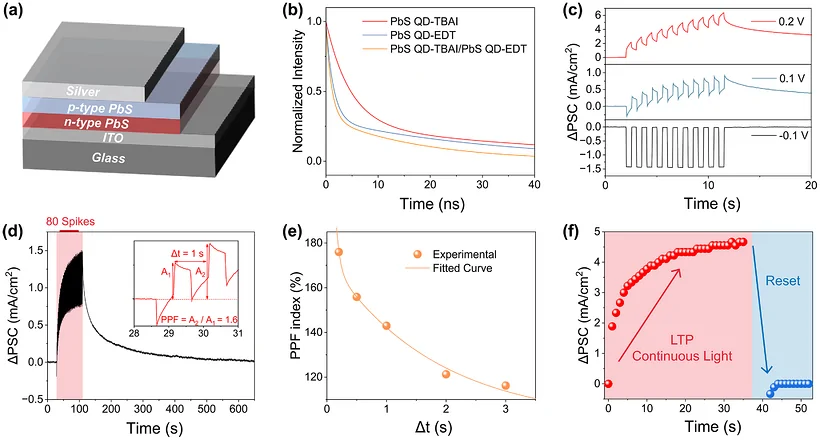
Characterization of PbS QD back‐to‐back rectifying junction synaptic devices. (a) Schematic illustration of the ITO/ PbS QD‐TBAI/PbS QD‐EDT/Ag device structure. (b) TRPL measurement for a single PbS QD‐TBAI layer (red) and PbS QD‐EDT layer (blue), and a bilayer PbS QD‐TBAI/PbS QD‐EDT homojunction (orange). (c) Measured bias‐tunable optoelectronic behaviors of the device under pulsed 808 nm illumination. (d) The PSC of the device in response to 80 pulses of 1 s duration, 808 nm light at 0.1 V bias. Inset: the definition of A_1_, A_2,_ and the PPF index. (e) Measured PPF index and fitting curve as a function of pulse time interval at 0.2 V bias. (f) LTP programming of the PbS QD synapse under continuous illumination of 808 nm light at 0.2 V bias and erasing/resetting of the PSC by −0.1 V bias.

Guided by the physical mechanisms revealed in our numerical simulations, we experimentally realized the back‐to‐back rectifying junction architecture using colloidal PbS QDs to verify its dual‐mode optoelectronic behavior. We fabricate layer stacks of PbS QD‐TBAI/PbS QD‐EDT /Ag on top of ITO/glass substrates (Figure 2a). One PbS QD‐TBAI and one PbS QD‐EDT layer are deposited sequentially on ITO by a layer‐by‐layer spin coating process in an ambient environment (see Methods) [33], followed by the thermal evaporation of Ag through a shadow mask as the top contact. AFM characterization of single‐layer PbS QD films confirms uniform and crack‐free morphologies before and after ligand exchange. PbS QD films become more densely packed after ligand exchange, and the surface roughness is reduced from 7.39 nm (as‐deposited) to 4.76 nm (PbS QD‐TBAI) and 4.48 nm (PbS QD‐EDT), respectively (Figure S6). Due to the proximity of the Fermi level of PbS QD‐TBAI (4.63 eV) to the work function of ITO (∼4.70 eV), the energy barrier at the ITO/PbS QD‐TBAI interface is trivial, and the electrical contact is considered nearly ideal ohmic [34]. Consequently, the device's optoelectronic behaviors are primarily governed by the back‐to‐back rectifying junction. The I‐V curves measured in the dark and under 808 nm illumination with varied powers match the simulation prediction (Figure S7), further validating the numerical model established above. We also conducted an experimental verification using control devices featuring either of the two individual junctions, corresponding to the simulation shown in Figure 1h. As shown in Figure S8, although the control device of PbS QD‐TBAI/ PbS QD‐EDT/Au exhibits a minor degree of synaptic plasticity, possibly due to charge traps acting as charge‐trapping centers, its synaptic behavior is significantly weaker. This comparison further confirms that while interfacial defects provide a minor contribution, the observed high‐performance and switchable synaptic behavior is intrinsically governed by the band alignment of the back‐to‐back junction architecture.

To verify the carrier dissociation efficiency of the PbS QD‐TBAI/PbS QD‐EDT homojunction, time‐resolved photoluminescence (TRPL) measurements are performed in air at room temperature. With 808 nm excitation, the TRPL spectra at 1550 nm of single PbS QD‐TBAI and PbS QD‐EDT layers and bi‐layer PbS QD‐TBAI/PbS QD‐EDT films (Figure 2b) can be fitted using a bi‐exponential decay function [35]:

$$I\left(t\right)=A_{1}\exp\left(\frac{-t}{\tau_{1}}\right)+A_{2}\exp\left(\frac{-t}{\tau_{2}}\right)$$

The radiative recombination of PbS QDs is associated with the lifetime of the slow decay component τ_2_. The fitted lifetime of the fast decay component τ_1 _is attributed to surface trap‐mediated recombination for individual PbS QD films treated by TBAI or EDT, while it reflects the charge separation facilitated by the built‐in electric field of the PbS QD‐TBAI/PbS QD‐EDT junction [36]. Compared to τ_1_ = 1.40 ns of the PbS QD‐EDT sample, the TBAI‐treated PbS QD film exhibits a longer τ_1_ = 4.58 ns, consistent with more efficient surface passivation by I^−^ ions [37]. In contrast, the PbS QD‐TBAI/PbS QD‐EDT junction yields the shortest τ_1 _= 1.08 ns, indicating significant PL quenching due to spontaneous charge separation and transfer at the homojunction interface.

The bias‐modulated optoelectronic characteristics of the PbS QD device are investigated under 808‐nm pulsed light irradiation, as shown in Figure 2c. Under a negative bias of −0.1 V, the device operates in a photodetector mode with instantaneous photoresponse and short rise/fall times, consistent with simulation results. Upon switching to a positive bias of 0.1 V, the device transitions into a synapse mode. When the positive bias is relatively low (<0.1 V), the photocurrent shows two spikes in opposite directions immediately after illumination on and off, whereas increasing V_bias_ to 0.2 V is akin to typical synaptic behavior. These two synapse regimes have been captured by the numerical model above as the synapse modes I and II, respectively.

However, the bias voltages required to transition between these regimes of practical samples do not perfectly align with the simulation. We attribute this discrepancy to a non‐ideal Schottky barrier and a weakened p‐n junction barrier height. As confirmed by temperature‐dependent *I–V* measurements, the actual *Φ_SC_* of the PbS QD‐EDT/Ag interface is reduced to ∼0.23 eV, compared to 0.6 eV used in the simulation, possibly caused by Fermi‐level pinning (Figure S9). Simultaneously, VB XPS measurements verify that ambient air exposure induces p‐type doping in the PbS QD‐TBAI layer [38] (Figure S10), which decreases *Φ_pn_* by 0.2 eV. Consequently, applied biases less positive than 0.3 V or less negative than −0.3 V are sufficient to modulate carrier transport across the back‐to‐back junction and switch operation modes. This behavior aligns well with the generalized correlation in Figure 1g, where a reduced *Φ_SC_* or a shift toward p‐type PbS QD‐TBAI systematically lowers the required mode‐switching voltages. A discrepancy between the simulated and experimental photocurrent magnitude is also noted, which is primarily attributed to the simplification of the model without involving material trap states and contact resistance that exist in the fabricated devices. However, since the bias‐dependent photoresponse and distinctive four‐regime synaptic behavior are well described in the simulation, the 2D numerical model still provides critical semi‐quantitative guidance for studying and designing the carrier dynamics of the back‐to‐back rectifying junction.

We evaluate the synaptic performance metrics at V_bias_ = 0.1 V (Figure 2d). The paired pulse facilitation (PPF) is defined as the amplitude ratio of the PSC peaks induced by two consecutive light pulses [39]:

$$PPF=A_{2}/A_{1}\times 100\%$$

where A_1_ and A_2_ are the magnitudes of the first and second PSC peaks, respectively. For a light pulse interval of 1.0 s, the PPF reaches 1.60 (Figure 2d). After 80 repeated pulses, the PSC accumulates to 1.5 mA/cm^2^ without reaching saturation, demonstrating high weight tunability for creating multi‐level conductance states for implementing synapse‐based neural networks. Furthermore, as shown in Figure 2e, the PPF index follows a double‐exponential decay as a function of the pulse interval time, with a maximum of 1.76 at 0.4 s. This behavior enables the solid‐state device to mimic the temporal dynamics observed in biological synapses for vision systems.

Another critical parameter is retention time, defined as the duration required for the PSC to decay to 10% of its peak value after illumination is removed. Here, 10% threshold is selected to avoid fluctuations in the dark current baseline. The retention time can be increased from 46 to 170 s by extending the number of optical pulses from 10 to 80, consistent with a transition from short‐term memory (STM) to long‐term memory (LTM). In addition to pulse numbers, this transition is also tunable by other parameters, including the bias magnitude (Figure 2c), pulse frequency, and light intensity (Figure S11).

The dynamic dual‐mode switching offers programmable photosynapse with high operational versatility. In this context, the “optical writing” (mimicking long‐term potentiation (LTP)) and “electrical erasing” (mimicking long‐term depression (LTD)) functions are realized by switching the operating voltage coupled with illumination conditions. As shown in Figure 2f, under a 0.2 V bias and continuous 808‐nm laser light, the device is written with optical information and memorizes it by trapping photogenerated carriers. To reset the current to its initial state, the bias is switched to −0.1 V with the optical excitation off, instantly sweeping out stored carriers. This cooperation between the synapse and photodetector modes through applied bias allows high‐speed in‐sensor processing of image data to effectively minimize data redundancy and enhance SNRs [40].

The back‐to‐back rectifying junction configuration is generally applicable to other material systems for generating synaptic plasticity. For instance, another intrinsically n‐type film consisting of 2D MoS_2_ nanoflakes can be deposited from their alcoholic dispersions and used to replace the PbS QD‐TBAI layer in the stack. The appropriate band edge positions yield similar synaptic behaviors with a PPF of 1.6 at a 1.0 s light pulse interval under V_bias_ = 0.1 V (Figure S12). Although MoS_2_‐based device exhibits an even higher PPF, MoS_2_ is not ultimately selected for further investigation because our solution‐processed MoS_2_ films fabricated from commercial inks suffer from poor morphological uniformity and reproducibility.

### 2.3 Wavelength‐Dependent Performance and Cascade‐Junction Strategy

**FIGURE 3:**
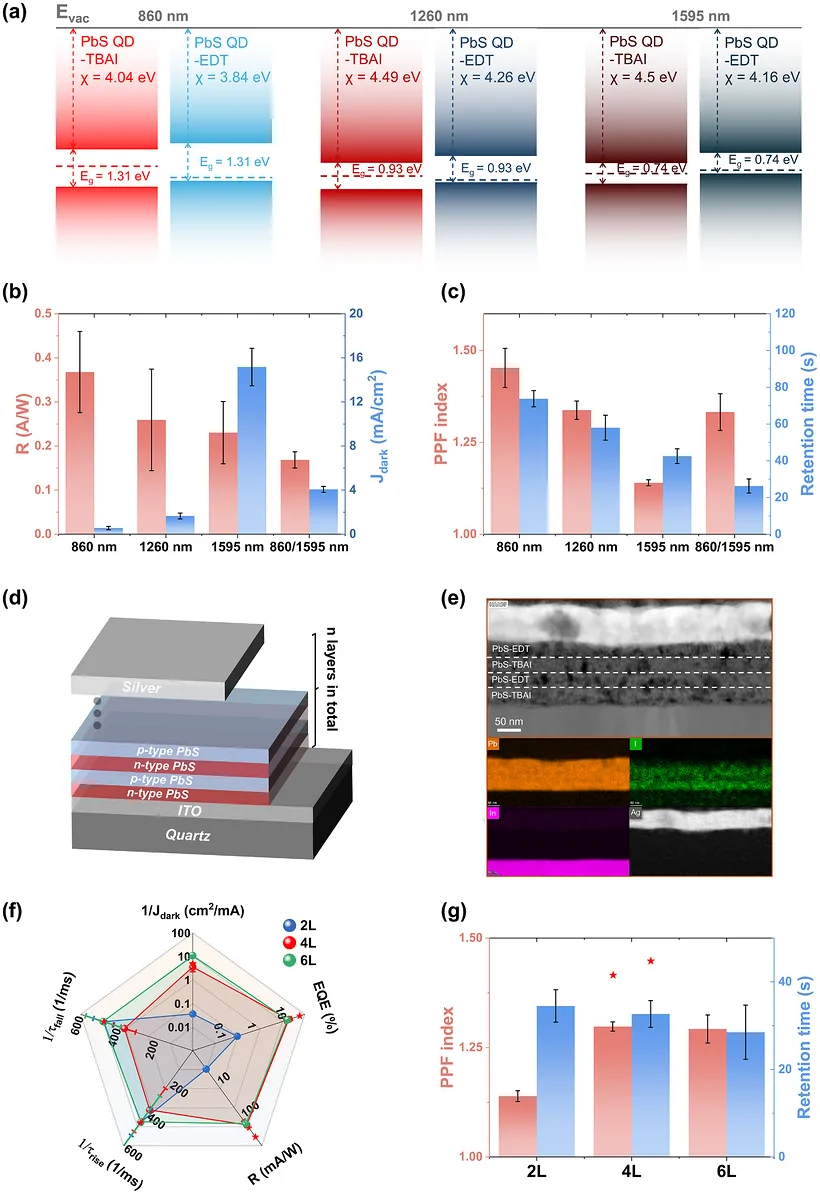
QD size‐dependent device performance and cascade‐junction strategies. (a) Band diagrams of PbS QD‐TBAI/PbS QD‐EDT homojunctions for 860, 1260, and 1595 nm PbS QDs before contact. Comparison of key performance metrics for 860, 1260, and 1595 nm PbS QD, and 860/1595 nm PbS QD devices in (b) photodetector (V_bias_ = −0.1 V) and (c) synapse (V_bias_ = 0.2 V) mode under 808 nm illumination pulsed at an interval of 1.0 s. (d) Schematic illustration of the cascade‐junction strategy by alternatively depositing PbS QD‐TBAI and PbS QD‐EDT layers. (e) TEM images of the cross‐section of the 4L cascade‐junction device and corresponding EDS mapping indicating the layered structure. Comparison of key performance metrics for 2L, 4L, and 6L configurations in (f) photodetector (V_bias_ = −0.1 V) and (g) synapse (V_bias_ = 0.2 6V) mode under 1550 nm illumination pulsed at an interval of 1.0 s. The star symbol denotes the champion device with the 4L structure and doubled thickness for each layer. The error bars represent standard deviations evaluated from four independently fabricated devices per condition.

Having established the bias‐switchable synaptic plasticity in standard bilayer QD junctions under NIR illumination, we next seek to extend the operating wavelength into the SWIR regime (>1500 nm). By utilizing the size‐dependent bandgap of PbS QDs, we can further modify the energy landscape of the device and engineer its photoresponse toward longer incident wavelengths. PbS QDs with varying sizes are synthesized, with diameters of 2.6 ± 0.12 nm, 4.3 ± 0.20 nm, and 5.9 ± 0.23 nm as determined by transmission electron microscopy (TEM, Figure S13). These sizes position their first excitonic absorption peaks at 860, 1260, and 1595 nm, respectively, spanning from the NIR to the SWIR region. For ease of associating each QD layer with its responsive spectral range, these materials are referred to by their excitonic absorption peak wavelengths throughout the remainder of this manuscript. To ensure the back‐to‐back rectifying junction alignment, band diagram parameters of their films treated by TBAI and EDT are extracted from VB‐XPS, UPS, and UV–vis Tauc plots (Figures S1 and S2). Results plotted in Figure 3a confirm that the staggered Type‐II homojunction is formed between the PbS QD‐TBAI and PbS QD‐EDT layers of the same QD size. Fourier‐transform infrared (FTIR) characterization confirms that the ligand exchange is thorough and essentially size‐independent across all QD films, ensuring no adverse impact on subsequent device comparisons (Figure S14 and Table S1). Then, three different‐sized QDs are used to fabricate PbS QD‐TBAI/PbS QD‐EDT/Ag multi‐junctions on ITO/glass to evaluate their photodetector and synapse performance under the same 808‐nm laser illumination, where these devices are denoted by the absorption peak wavelength of the constituent PbS QDs. Operating as a typical photodetector at V_bias_ = −0.1 V, devices of larger QD sizes with narrower bandgaps lead to a degradation in both responsivity (R) and dark current density (J_dark_) (Figure 3b). Specifically, R drops from 0.37 to 0.26, and to 0.23 A/W, while J_dark_ increases from 0.53 to 1.62, and to 15.1 mA/cm^2^, as the QD absorption peak shifts from 860 to 1260 nm and to 1595 nm. This could be due to increased thermal generation of carriers in the dark and lowered energy barriers for inefficient charge separation as the PbS QD bandgap narrows [41], and more prevailing defects and unpassivated surfaces in larger PbS QDs [42].

In the synapse mode with V_bias_ = 0.2 V, both the PPF index and retention time decrease with the bandgap for the same 808 nm laser pulses (Figure 3c). The PPF index and retention time of 1595 nm PbS QD devices drop to 1.14 and 42.5 s, while those for 860 nm PbS QD devices are 1.46 and 73 s, respectively. As shown in Figure 3a, the Fermi level of the PbS QD‐EDT layer shows weaker dependence on QD size, leading to similar *Φ_sc_* across these devices. Meanwhile, *Φ_pn_* of the QD homojunction is substantially reduced as the QD size enlarges, which is the major cause of the inferior carrier trapping performance, also consistent with the trend of photodetector metrics above. This comparison suggests that extending the response wavelength to >1500 nm into SWIR by simply increasing the QD size is insufficient for maintaining high‐performance dual‐mode detection.

As a strategy to improve the potential barrier between two QD layers, we replace 1595 nm PbS QDs with 860 nm PbS QDs to construct the TBAI‐treated layer and interface with the 1595 nm PbS QD‐EDT/Ag junction (860/1595 nm device). By doing so, while J_dark_ is successfully reduced to 4.06 mA/cm^2^, the R of photodetector mode reaches the lowest value of 0.17 A/W, suggesting hampered charge separation efficiency (Figure 3b). TRPL decay is not enhanced by forming the 860/1595 nm PbS QD junction (Figure S15), consistent with the formation of a Type‐I heterojunction between the 860 nm PbS QD‐TBAI and 1595 nm PbS QD‐EDT layers based on their energy band levels. Nevertheless, this QD size engineering strategy favors carrier trapping. As a synapse, the 860/1595 nm device shows improved PPF by 0.19 compared to the one made entirely of 1595 nm PbS QDs (Figure 3c).

The back‐to‐back rectifying junction concept can be extended from two PbS QD layers to more generalized cascadejunction configurations by alternately depositing PbS QD‐TBAI and PbS QD‐EDT layers. The solid‐state ligand exchange treatment ensures stacking of distinctive ligand‐capped QD films in the layer‐by‐layer spin coating approach [43]. Akin to the Type‐II superlattice strategy employed in epitaxial IR photodetector systems for suppressing dark current [44], we expect the cascade QD junctions to also enhance the device performance under the SWIR wavelengths. Herein, multi‐junction devices are fabricated with one to three units of PbS QD‐TBAI/PbS QD‐EDT bilayers, corresponding to two to six layers (L) of QD films, respectively, consisting solely of 1595 nm PbS QDs (Figure 3d). To ensure consistency of the total QD film thickness, the concentration of PbS solutions is diluted from 50 to 25 mg/mL and to 17 mg/mL, as the number of QD layers increases from 2 to 6 [45]. As shown in Figure S16, atomic force microscopy (AFM) confirms that uniform and smooth QD films with a total thickness of 93 ± 8 nm are achieved even though the QD film varies from 2 to 6L of repetitive PbS QD‐TBAI and PbS QD‐EDT deposition.

In the photodetector mode under 1550 nm illumination, the 4L cascade‐junction device achieves a notable 100‐fold suppression of J_dark_ compared to the 2L cascade‐junction devices, decreasing from 15.1 to 0.277 mA/cm^2^, and further increasing to 6L reduces J_dark_ to 0.0918 mA/cm^2^ (Figure 3f). This suggests periodic potential wells defined by the layer interfaces effectively impede transport of thermally generated carriers in the dark. Furthermore, the thickness of each QD layer partitioned by the multi‐junction structure (less than 50 nm) is thinner than the carrier diffusion length, thus reducing recombination of photogenerated carriers. This leads to an enhancement in R by increasing the number of cascade junctions from one to three, achieving an optimal 184 mA/W in the 4L cascade‐junction device (EQE = 14.7%). However, a slight degradation is observed in the 6L cascade‐junction device, where R drops to 168 mA/W; this is constrained by the single‐layer thickness, which will be discussed in detail later. Furthermore, creating multi‐junction structures does not necessarily retard the response speed, as µs‐fast response is observed across all samples (between 1.6 and 3.5 µs). Specifically, the 6L cascade‐junction device exhibits the shortest average rise time (τ_rise_) of 2.3 µs and fall time (τ_fall_) of 2.1 µs.

Operated as optoelectronic synapses, as shown in Figure 3g, the 4 and 6L cascade‐junction devices exhibit a higher PPF (1.30) compared to the 2L cascade‐junction device (1.14), consistent with strengthened carrier trapping by cascading junctions. However, we notice that the reproducibility of the 6L cascade‐junction device is worsened, possibly due to overly reduced thickness (∼15 nm) of every PbS QD layer. Such a layer only contains 2–3 monolayers of 1595 nm PbS QDs, which may not be sufficient to compensate for the volume shrinkage during ligand exchange and leaves behind cracks. Also, the thin QD layer cannot block diffusion of ligand reagents effectively, causing additional and undesirable surface treatment on the underneath QD layer and altering its doping concentration as well as the band alignment of the cascade junction. In addition, we observe a decrease in the averaged retention time for 6L cascade‐junction devices, as the downward band bending in the PbS QD‐EDT layer is flattened when the depletion regions extend from both sides of the junctions into the thin QD layer and merge to lower the potential barriers for hole trapping. Comparative simulations (Figure S17) demonstrate such phenomena, where the *Φ_pn_* within a PbS QD‐TBAI/PbS QD‐EDT/PbS QD‐TBAI unit decreases as the single‐layer thickness reduces. Consequently, from the perspective of optoelectronic characterization, for a fixed two‐layer device, increasing the single‐layer thickness improves its synaptic effect (Figure S18). Therefore, our simulations suggest that preserving a sufficient individual layer thickness is necessary to maintain the interfacial energy barriers for trapping carriers in synapse mode and separating carriers in photodetector mode.

Guided by this simulation result to further facilitate IR light absorption and optimize device performance based on 4L device, the single‐QD‐layer thickness and thus the total absorber thickness of the 4L device are doubled by utilizing a 50 mg/mL PbS QD solution during spin‐coating. The thickened 4L device has the same single‐layer thickness as that of the 2L device but shows significantly enhanced photodetector responsivity and synaptic plasticity. These observations suggest potential performance enhancements can be achieved by increasing the number of stacking layers with constant individual layer thickness. The distinct layered structure of this device cross‐section is verified via high‐resolution TEM imaging (Figure 3e), where EDS elemental mapping clearly confirms the presence of four alternating PbS QD‐EDT and PbS QD‐TBAI layers between the bottom ITO/glass and top Ag electrodes within a total QD film thickness of approximately 140 nm. The champion device (denoted by the star symbol in Figure 3f,g) with these thicker QD layers exhibits J_dark_ = 0.199 mA/cm^2^, R = 430 mA/W, and EQE = 34.4% under −0.1 V and 1550 nm illumination. Notably, this performance is obtained in the absence of an electron‐transporting ZnO layer, which is typically necessary for high‐performance PbS QD IR photodiodes [44, 46], demonstrating effective charge separation of the cascade junction design. Moreover, the device exhibits a highly linear photocurrent response over a wide optical intensity range from 100 µW/cm^2^ to 500 mW/cm^2^ (Figure S19). The device does not compromise the response time, still maintaining a t_rise_ and t_fall_ of 2.2 and 2.1 µs, respectively, which corresponds to an estimated equivalent 3‐dB bandwidth (*f*_‐3 dB_ ≈ 0.35/t) of approximately 159 kHz. In response to pulsed 1550 nm laser light, the 4L cascade‐junction device with increased single‐layer thickness shows improved synaptic PPF of 1.41 and retention time beyond 43 s. The full dataset of this champion device is included in Figure S19. Furthermore, we evaluated the long‐term stability of the champion device stored in the ambient environment over an extended period (Figure S20). Due to oxidation of the QD film with degraded conductivity and the built‐in electric field, the device experiences initial performance decay over the first two days, after which the degradation gradually decelerates and reaches a stabilized saturation state. Device encapsulation is thus needed for long‐term operation in air. The performance enhancement demonstrated by this optimized device underscores the potential of solution‐processed cascade‐QD‐junctions as a practical strategy for achieving efficient SWIR neuromorphic vision systems.

### 2.4 Switchable Photodetector and Synapse Modes Enabling Multi‐Functional IR Vision Sensing

To translate the enhanced SWIR photodetection and synaptic plasticity of our cascade‐junction device into system‐level sensory applications, we evaluated its utility in computing tasks and neuromorphic vision processing. For IR images with low SNRs, denoising can be a critical pre‐processing step to enhance the contrast for the following object recognition. The history‐dependent, accumulative synaptic PSC can be utilized to suppress noise signals. We intentionally introduce Gaussian noise to the images of handwritten digits in the MNIST dataset and simulate their projection on the multi‐junction PbS QD device arrays with the same resolution. By encoding the brightness of each pixel to the pulse duration locally illuminated on the corresponding synapse device, we obtain sustained PSC in the desired feature areas and quickly decaying PSC at the low‐brightness noise pixels. Consequently, the recognition accuracy of the handwritten digits by an artificial neural network (ANN) is enhanced by 30% through the synapse denoising process. Detailed description and results of this simulation are provided in Figure S21. Therefore, the synapse mode of the multi‐junction device serves as a high‐pass filter and effectively removes background noise at the hardware level. This bio‐inspired mechanism mimics the initial signal modulation in the human visual system, where peripheral neurons filter out unnecessary stimuli before higher‐level processing to enhance sensory data quality [47].

**FIGURE 4:**
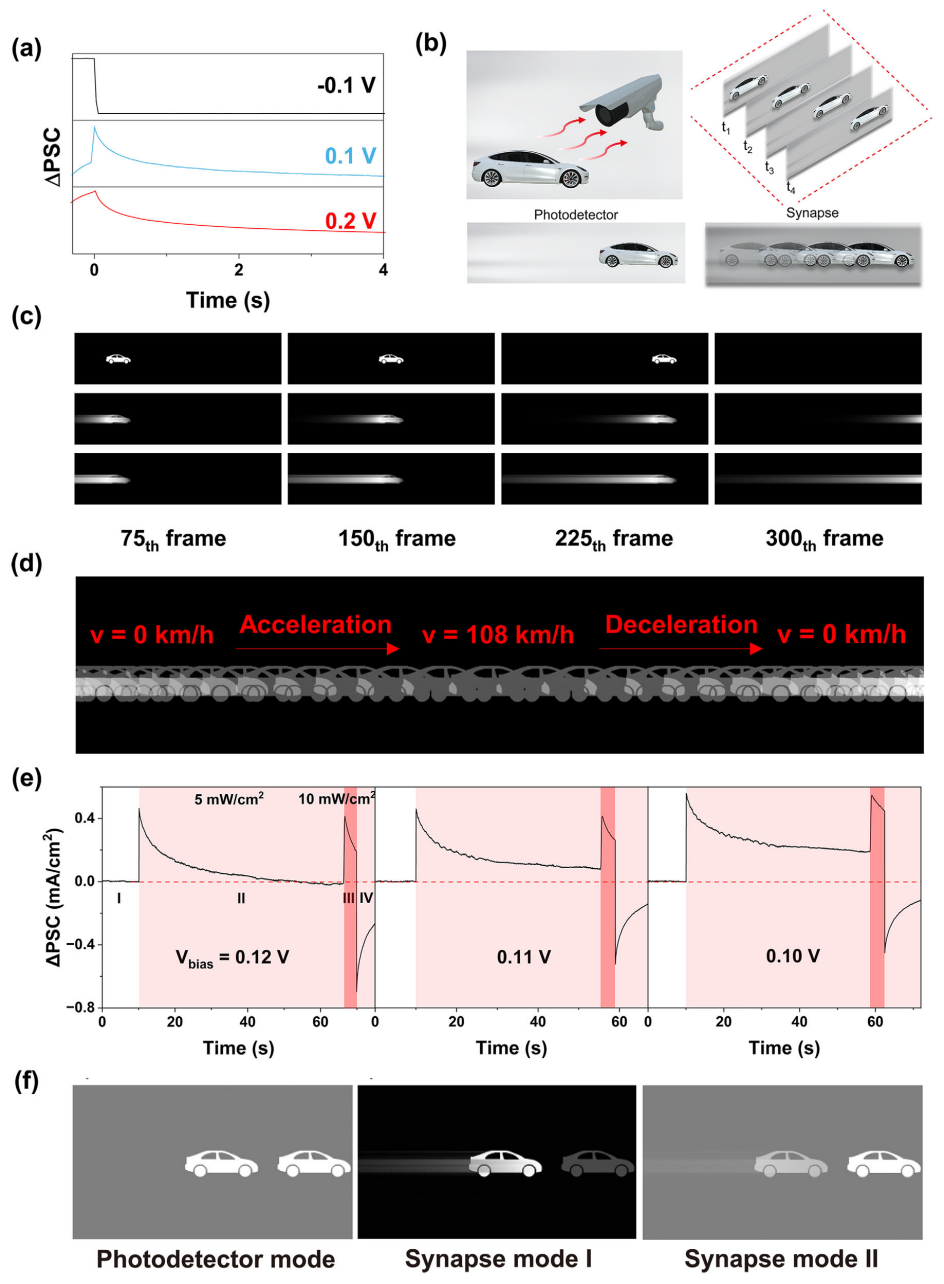
Simulation of SWIR motion trajectory tracking and hardware‐level static background suppression. (a) Modulation of relaxation time (τ) of the PSC generated by multi‐junction PbS QD device via applied bias. (b) Schematic diagram of dual‐mode vision sensors for simultaneous real‐time positioning and continuous trajectory tracking. (c) Representative frames illustrating real‐time position (top row) vs. spatiotemporal trajectory tracking (bottom two rows) at −0.1, 0.1, and 0.2 bias levels (from top down), respectively. (d) Motion history reconstruction of a car moving from left to right, while accelerating from 0 to 108 km/h and decelerating to a stop. (e) The measured PSC response of a single‐pixel device with static background photocurrent suppression by precisely tuning bias under stepped light intensities from 5 mW/cm^2^ (baseline) to 10 mW/cm^2^ (stimulus). (f) Simulation comparison of SWIR imaging performance among three operational modes.

Beyond preprocessing static signals, the synaptic device behavior also provides the temporal resolution for identifying dynamic targets. The photosensitive excitation and inhibition of the synaptic response allow for the reconstruction of the motion trajectory [14, 15, 16]. Meanwhile, dynamic switching to the photodetector mode can reset the imaging baseline and capture the static environment. Specifically, the temporal dynamics are characterized by the relaxation time τ [16]. In synapse mode, τ is defined as the duration for the PSC to decay to 1/e of the peak value, while in photodetector mode, τ is the fall time. As shown in Figure 4a, the device exhibits a widely controllable τ by the applied bias. Such tunable temporal dynamics are essential for detecting moving speeds from images taken under synapse mode.

In the simulated scenarios, an array of SWIR optoelectronic sensors (1600 × 400) constructed from the multi‐junction PbS QD device parameters monitors the horizontal motion of a car driving from left to right (Figure 4b), where the 10‐s movement is partitioned into 300 temporal frames. Assuming a dark background and a “bright” object with reflected SWIR light, at each frame, the pixels corresponding to the transient position of the target car are illuminated, while the other area of the sensor array is in the dark. Therefore, the total signal received by each pixel is the integration of the instantaneous photocurrent generated by the local device from time zero to the current time. The spatiotemporal motion of the car is encoded into the brightness of the SWIR image captured by the sensor array. Four snapshots at the 75th, 150th, 225th, and 300th frames are extracted to compare how the device's dynamic photoresponse property shapes the characteristics of the output image (Figure 4c). At V_bias_ = −0.1 V, the multi‐junction PbS QD device works in the photodetector mode, and the rapid photoresponse allows the system to capture the precise real‐time position of the car. When operated in the synapse mode, every device pixel produces photocurrent with retention, resulting in a tail following the blurred car image as the motion trajectory. The trajectory tail can be elongated, as V_bias_ increases from 0.1 to 0.2 V, and τ extends from 0.9 to 3.5 s. This motion tracking image contains information on both the moving direction and velocity. For instance, Figure 4d presents a picture taken under the synapse mode, where a car drives from left to right, accelerating from a stationary state to 108 km/h and then decelerating to a full stop. The image with residue traces is a physical record of the motion history, and the brightness of the feature is inversely proportional to the car speed. This demonstration indicates that the temporal information can be condensed into a single‐frame image, significantly reducing data volume compared to conventional video‐based monitoring and enabling efficient, low‐bandwidth spatiotemporal perception.

However, achieving the above high‐contrast motion tracking relies on the assumption of a perfectly dark background. In fact, background noise poses a more significant challenge for history‐dependent synaptic imaging than for conventional photodetectors with instantaneous response. Consequently, for high‐speed objects, the dwell time on a single synapse pixel is too short to generate a strong PSC and form a sufficient SNR when competing against the steady contribution of the static background noise. For instance, as shown in Figure S23, as the illumination light intensity doubles from 5 to 10 mW/cm^2^, the transient PSC of the synapse (V_d_ = 0.5 V) only increases by 48%, while the photocurrent magnitude linearly follows the light intensity in the photodetector mode (V_d_ = −0.5 V).

A niche neuromorphic vision sensor design can suppress the static background noise, namely, DVS. The core principle of a DVS is known to generate a signal spike only triggered by a change in light intensity but filter out static signals. This asynchronous photoresponse is not directly proportional to the absolute light intensity. Interestingly, our multi‐junction device exhibits DVS‐like behaviors by carefully tuning the applied bias in the synapse mode I, as shown in Figure 4e. The experimental setup for a single‐pixel proof‐of‐principle test is illustrated in Figure S22, where a light source of 10 mW/cm^2^ is used to simulate the signal reflected from a moving target, while the other light source is set at 5 mW/cm^2^ as static background. A sudden increase in light intensity, as the target object passes by this device, induces a transient photocurrent spike. As discussed previously, photogenerated electron current in this regime can be counteracted by the de‐trapped holes. By applying a bias of 0.12 V experimentally, we achieve a nearly zero net baseline current even under a constant 5 mW/cm^2^ background illumination. As a result, the SNR of the non‐linear photoresponse spike, I_ph_ (10 mw/cm^2^)/I_ph_ (5 mw/cm^2^), becomes as high as 30. This result highlights another advantage of bias‐dependent synaptic characteristics and promises the multi‐junction QD device for DVS imaging applications. Additionally, COMSOL simulation of the identical process shows consistent results with the experimental observations, as summarized in Figure S23.

Based on this mechanism, the final reconstructed images are presented in Figure 4f, including a moving car on the left side and a stationary car parked on the right for comparison. At synapse mode II, the signal intensity of the moving car is lower than that of the stationary car. With low imaging contrast and an unidentifiable motion trajectory, the moving car is submerged in the background noise. In contrast, when reducing the bias to baseline‐suppressed synapse mode I, the signal from the moving car becomes significantly enhanced, the trajectory tail is clearly observed, and the separation from the standstill car is distinct. Meanwhile, the floor signal is greatly suppressed to achieve high SNRs. In sum, our multi‐junction PbS QD device design not only outperforms reported dual‐mode devices in the SWIR regime, but also remains competitive when evaluated against single‐mode PbS QD photodetectors with optimized transport layers.  The tunable synapse mode offers an operation regime that filters out redundant stationary signals, while selectively capturing dynamic objects with their historical motion information.

## 3 Conclusions

In summary, a multifunctional optoelectronic platform based on a back‐to‐back rectifying junction has been theoretically designed and experimentally realized by PbS QD devices for SWIR neuromorphic vision sensing. By engineering the PbS QD‐TBAI/PbS QD‐EDT/Ag structure that creates a potential well for photogenerated holes within the p‐type PbS‐EDT layer, the device exhibits a reconfigurable transition between a high‐speed photodetector mode and a persistent synapse mode. The operation mode is switchable by modulating the barrier heights of the back‐to‐back junctions through applied bias. QD size engineering and cascade‐junction stacking are introduced to mitigate the inherently low barrier heights of small‐bandgap PbS QDs for enhanced SWIR photodetection and sensing. The optimized 4L cascade‐junction device demonstrates an R of 430 mA/W and EQE of 34.4% under 1550 nm illumination, while suppressing J_dark_ to 0.199 mA/cm^2^ as a photodetector. Applying a positive V_bias_, a PPF of 1.41 and an extended retention time of 43 s are achieved in synapse mode. These device characteristics can encode optical signals to instantaneous or persistent photocurrent, leading to applications in neuromorphic vision for SWIR spatiotemporal perception, including image denoising, motion trajectory tracking, and static background suppression. The bias‐tunable mode‐switching capability not only enables reconstruction of high‐fidelity motion trajectory in a single frame but also resolves a critical bottleneck of low SNRs in synapse‐based SWIR imaging. As benchmarked against state‐of‐the‐art dual‐mode image sensors (Table S2), our cascade architecture demonstrates distinct advantages in achieving SWIR spectral coverage (>1500 nm), dynamic mode‐switching, and DVS‐like output with high SNR. Notably, since the functional transition between detection, memory, and event‐based filtering is governed solely by a unified device physics mechanism, this multifunction device significantly reduces circuit complexity and provides versatility for further integration. Co‐designed with adaptive algorithms and operational models, this solution‐based reconfigurable vision system will provide a scalable path toward high‐dimensional vision fusion and intelligent SWIR sensing in complex environments.

## 4 Methods

### 4.1 Materials

Pb(Ac)_2_·3H_2_O (Aladdin, 99.99%), bis(trimethylsilyl)sulfide ((TMS)_2_S; Aldrich, 98%), NaAc (TCI, ≥98.5%), oleic acid (OA; Macklin, analytical reagent grade [AR]), 1‐octadecene (ODE; Aladdin, 90%), dimethylformamide (DMF; Macklin, 99.5%), octane (Aladdin, 96%), butylamine (BA; Aladdin, 99.5%), acetone (VWR, AR), Tetrabutylammonium Iodide (TBAI; Aladdin, 99%), 1,2‐ethanedithiol (EDT; Aladdin, ≥98% GC), acetonitrile (ACN; Anaqua, AR), and methanol (MeOH; Anaqua, AR).

### 4.2 PbS QD Synthesis

PbS QDs are synthesized using the method reported by Hines and Scholes, with minor adjustments [48]. For QDs with a peak absorption of 1595 nm and ∼6 nm diameter, Pb(Ac)_2_·3H_2_O (0.765 g), OA (18 mL), and ODE (10 g) are added into a 250‐mL three‐necked flask and degassed at 110°C for 2 h. When the Pb precursor is heated to 120°C, the S precursor ((TMS)_2_S, 210 µL in 10 mL of ODE) is swiftly injected into the flask, and the heater is subsequently removed. As the solution naturally cools to room temperature, 30 mL of acetone is poured into it and stirred for 1 min, followed by centrifugation at 4000 rpm for 5 min. The resulting precipitate is purified through three cycles of redispersion in 6 mL of octane and precipitation with 20 mL of acetone. Finally, the purified precipitate is dried under vacuum at room temperature for 30 min and redispersed in octane to form a 50 mg/mL PbS QD solution. For QDs with absorption peaks of 1260 and 860 nm, the amount of OA is adjusted to 9 and 1 mL, respectively, while other parts of the procedure remain unchanged.

### 4.3 Device Fabrication

The PbS QD optoelectronic synapses are fabricated as follows: ITO/glass substrates are cleaned via sequential ultrasonication in acetone, isopropyl alcohol, and deionized (DI) water, followed by a 20 min UV‐Ozone treatment. The PbS QD layers are then fabricated onto the ITO substrates by layer‐by‐layer spin coating. One PbS QD‐TBAI layer is deposited by spin‐coating the QD solution at 1500 rpm for 30 s. The solid‐state ligand exchange is achieved by soaking the as‐prepared film in 10 mg/mL TBAI methanol solution for 40 s and rinsing with pure methanol. For the PbS QD‐EDT layer, an EDT solution (0.02 vol% in ACN) is applied to the spin‐coated PbS QD films for 30 s followed by an ACN rinsing step [49]. This procedure is repeated to achieve the desired number of layers. The films are then annealed at 80°C for 20 min. All spin coating and heating processes are conducted in air with humidity below 20%. Finally, Ag electrodes (100 nm thick) are thermally evaporated on top of the films through a shadow mask at a base pressure of 10^−5^ mbar.

### 4.4 Material Characterization

The VB‐XPS and UPS spectroscopy of PbS QD films is measured by a Thermo Scientific Escalab QXi X‐ray Photoelectron Spectrometer Microprobe, and a sample bias of −10 V is applied for UPS acquisition. The absorption spectrum of PbS QDs is collected with an Agilent Cary 5000 UV‐vis‐IR spectrophotometer. The TRPL measurement of PbS QD films is obtained by a OmniFluo‐900 Steady‐State & Time‐Resolved Fluorescence Spectrometer. The top‐view SEM image of PbS QD film is acquired using Leo 1530 FEG SEM. The AFM image of PbS QD film is characterized by Bruker JPK NanoRacer/ NanoWizard 4 XP AFM. The cross‐section image of the device is prepared by slicing layered samples using FEI Quanta 200 3D FIB, and then imaged on a Thermo Scientific Talos F200X STEM.

### 4.5 Device Characterization

The optoelectronic performance of the PbS QD optoelectronic synapses is characterized using specialized measurement configurations tailored to the illumination wavelength and temporal resolution. For 808 nm light illumination, the source is a FU808AD100‐GD16 laser. Light intensity is calibrated using an optical power meter (PM100D). A GCI‐73 M electronic timer/shutter is used to regulate the duration of light pulses. The current‐voltage and current‐time characteristics are measured using a Keysight B1500A semiconductor parameter analyzer. For 1550 nm light illumination, the source is an MDL‐III‐1550‐100 mW laser. Light intensity is calibrated using a Ge photodetector (FDG03). The current‐voltage and current‐time characteristics are measured using a Keithley 2400 source meter. For response time measurement, a 940 nm LED is used as the optical pulse source, and the pulse signal is applied using a function generator (AFG31000). The response‐time signal is first amplified using a transimpedance amplifier (SR570) and then measured with an oscilloscope (TBS2000B). After the I‐V measurements, the EQE and R are calculated using the equations as follows,

$$R=I_{ph}/P$$

where *I_ph_* is the photocurrent and *P* is the incident power of the illumination.

EQE=Rhc/qλ

where h is Planck constant, c is the speed of light, q is the elementary charge, and λ is the wavelength of the illumination.

### 4.6 COMSOL Modeling

The back‐to‐back rectifying junction is simulated in a 2D model using COMSOL Multiphysics 6.3 (Figure S24). A two‐layer geometry representing the stacked PbS QD‐EDT and PbS QD‐TBAI films is constructed. The Semiconductor module is utilized to solve for carrier transport, while the Electromagnetic Waves module is applied to compute the optical absorption profile; these two modules are coupled to account for carrier photogeneration. Furthermore, a transient solver is employed to investigate the temporal carrier dynamics. Notably, material carrier traps are intentionally omitted from the simulation. The fundamental material parameters describing the QD layers and the Schottky contact boundary conditions are summarized in Table S3. The numerical solutions are considered converged when the relative tolerance fell below 1 × 10^−^^6^.

### 4.7 Simulation of Motion Trajectory Tracking

Motion trajectory tracking is simulated across a 1600 × 400 pixel array using a Python script. To generate the dynamic target motion for Figure 4c, synthetic video frames are generated at 30 frames per second (fps), where each pixel holds a binary value (1 or 0) representing the localized presence or absence of illumination. Each pixel in the array functions as an independent optoelectronic synapse governed by parameters measured from real PbS QD devices. Under localized illumination (binary value 1), active pixels produce PSC via pulse‐dependent facilitation, whereas non‐illuminated pixels (binary value 0) undergo exponential decay governed by the time constant τ. The resulting spatiotemporal PSC matrices are normalized to grayscale frames to reconstruct motion trajectories.

To demonstrate the effect of the vehicle speed change in synapse imaging mode (Figure 4d), we define the motion of the vehicle passing through the window as follows: starting from rest, the vehicle accelerates uniformly at 10 m/s^2^ to 108 km/h, then decelerates uniformly at 10 m/s^2^ back to rest. The entire process takes 3 s, and the total distance that the vehicle travels is 90 m. The video frame rate is set to 10 fps, while other settings remain unchanged as above.

For the static background suppression exercise shown in Figure 4f, the binary values of pixels in the video represent different illumination densities corresponding to the steady background and dynamic event, the resulting PSC behaviors are determined by parameters derived from simulation, while other settings remain unchanged as above.

## Author Contributions

Z.H. and T.Z. conceived the idea and wrote the manuscript. Z.H. performed the COMSOL modeling, Python simulations, device fabrication, and preliminary optoelectronic characterization. D.O. measured the optoelectronic performance of the fabricated devices in detail. Y.J. and Y.L. synthesized the QD used in the device fabrication. C.S. assisted with the TRPL measurements and temperature‐dependent I‐V characterization. T.Z. and S.O. co‐supervised the project. All authors have edited and agreed on the manuscript.

## Conflicts of Interest

The authors declare no conflicts of interest.

## Supporting information




**Supporting File**: advs77617‐sup‐0001‐SuppMat.docx.

## Data Availability

The data that support the findings of this study are available from the corresponding author upon reasonable request.
