# Supplementary material for: Solution‐Processed Multi‐Junction Optoelectronic Synapses for Mode‐Switchable SWIR Neuromorphic Vision Sensing
Source: Adv Sci (Weinh). 2026 Sep 27:e77617. Online ahead of print. doi: 10.1002/advs.77617 (PMC13616316; doi:10.1002/advs.77617)
Supplement: Supplementary file 1 [file ADVS-9999-e77617-s001.docx]

**Supplementary Information**

**Solution-Processed Multi-junction Optoelectronic Synapses for Mode-switchable SWIR Neuromorphic Vision Sensing**

*Zhuoyang He^1^, Daeyang Oh^3^, Yi Ji^1^, Yanyan Li^1^, Chengwei Shan^1^, Soong Ju Oh^3*^, Tianshuo Zhao^1,2*^*

*^1^Department of Electrical and Computer Engineering, The University of Hong Kong, Hong Kong SAR, China.*

*^2^Materials Innovation Institute for Life Sciences and Energy (MILES), HKU-SIRI, Shenzhen 518045, China.*

*^3^Department of Materials Science and Engineering, Korea University, Anam-Dong, Seongbuk-Gu, Seoul, Republic of Korea.*

Email: [sjoh1982@korea.ac.kr](mailto:sjoh1982@korea.ac.kr), [tszhao@hku.hk](mailto:tszhao@hku.hk)

**Figure S1.** Valence band X-ray and ultraviolet photoelectron spectroscopy (VB-XPS and UPS) of three different-sized PbS QD films treated by tetrabutylammonium iodide (TBAI) and 1,2-ethanedithiol (EDT) solutions. PbS QD films are denoted by their absorption peak wavelengths. (a) VB-XPS spectra. (b) Fermi edge and (c) cut-off edge of UPS spectra under -10 V bias.

**Figure S2.** Tauc plots and derived bandgaps of three different-sized PbS QD films.

The band diagram of PbS QDs can be calculated by combining Ultraviolet-Visible spectroscopy (UV-Vis) (**Figure S2**), VB-XPS (**Figure S1a**), and UPS spectra (**Figure S1b and c**), which provide bandgap, VB edge, and Fermi level of PbS QD films, respectively. The Fermi level is calculated by:

$$E_{F}=21.22-(E_{cut-off}-E_{f})$$

The calculated E_f_ (eV) vs. vacuum is as follows:

|  | **860 nm** | **1260 nm** | **1595 nm** |
| --- | --- | --- | --- |
| PbS QD- EDT | 5.01 | 5.09 | 4.76 |
| PbS QD-TBAI | 4.64 | 4.96 | 4.88 |

**Figure S3.** Simulated energy band diagram of the PbS QD-TBAI/PbS QD-EDT/Ag structure at zero bias under dark conditions.

**Figure S4.** Simulated dark I-V characteristics of the PbS QD-TBAI/PbS QD-EDT/Ag device. The inset shows equivalent circuit diagrams for regions I, II, and III.

**Figure S5.** Comparison of postsynaptic current (PSC) characteristics and corresponding band diagrams of the PbS QD-TBAI/PbS QD-EDT/Ag device at (i) 0.1 V and (ii) -0.1 V bias voltage. Arrows represent the carrier transfer direction, with solid arrows representing the dominant direction.

**Figure S6.** Atomic Force Microscope (AFM) topographical images and surface roughness data for PbS QD films (a) before ligand change and (b) with TBAI ligand exchange and (c) with EDT ligand exchange.

**Figure S7.** Measured I-V characteristics of the PbS QD-TBAI/PbS QD-EDT/Ag device (a) in the dark and (b) under 808 nm light illumination with different light intensities.

**Figure S8.** Experimental photocurrents of the PbS QD-TBAI/PbS QD-EDT/Ag, PbS QD-EDT/Ag, and PbS QD-TBAI/PbS QD-EDT/Au devices under 808 nm light illumination at 0.1 V bias.

**Figure S9.** (a) Device structure of the Ag/PbS QD-EDT Schottky junction device used for temperature-dependent I-V measurements. (b) I–V characteristics of the device at various temperature. (c) Plot of ln[J(1-e^-qV/kT^)] versus V at different temperatures. (d) Plot of ln(J_0_/T^2^) versus T^-1^, where J_0_ is the saturation current density. *Φ_sc_* is extracted to be 0.23 eV.

By fitting the temperature-dependent I-V curves with

$$J=J_{0}[\exp(\frac{qV}{nk_{B}T})-1]$$

and $J_{0}=A^{*}T^{2}\exp\left(-\frac{q\Phi_{sc}}{k_{B}T}\right)$

, where *J_0_* is the saturation current density, *q* is the elementary charge (1.602×10^-19^ C), *V* is the applied bias voltage, *k_B_* is the Boltzmann constant, *T* is the absolute temperature, and n is the ideality factor. We experimentally extract the *Φ_sc_* as 0.23 eV, significantly lower than the theoretical expectation.

**Figure S10.** VB-XPS spectra of PbS QD-TBAI films as fabricated (bottom) and after air exposure for 12 hours (top).

**Figure S11.** (a) The PSC response of the PbS QD-TBAI/PbS QD-EDT/Ag device under 10 consecutive light pulse excitations with various light intensities of 5 mW/cm^2^, 10 mW/cm^2^ and 30 mW/cm^2^, exhibiting the transition from short-term memory (STM) to long-term memory (LTM) behavior. (b) The PSC response induced by a series of light pulses with varying frequencies within a 10-second interval.

**Figure S12.** (a) The PSC of the MoS_2_/PbS QD-EDT device measured at 0.1 V bias under 808 nm light pulse illumination.

**Figure S13.** Transmission Electron Microscope **(**TEM) images of PbS QDs with different sizes. TEM images of (a) 2.6-nm-diameter (absorption peak at 860 nm), (b) 4.3-nm-diameter (absorption peak at 1260 nm), and (c) 5.9-nm-diameter (absorption peak at 1595 nm) PbS QDs.

**Figure S14.** Fourier Transform Infrared Spectroscopy **(**FTIR) spectra of EDT- and TBAI-capped PbS QD films across different particle sizes ((a) 860 nm, (b) 1260 nm, and (c) 1595 nm absorption peaks). Shaded areas highlight the specific vibrational bands used to quantify the ligand exchange efficiency reported in Table S1.

**Table S1. Size-dependent Ligand exchange efficiency of PbS QD films.**

**(Normalized to the integration of C-H intensities of 860 nm PbS QDs as 100%)**

|  | **860 nm** | **1260 nm** | **1595 nm** |
| --- | --- | --- | --- |
| PbS QD- EDT | 100% | 128% | 88% |
| PbS QD-TBAI | 100% | 96% | 98% |

**Figure S15.** (a) Transient photoluminescence (TRPL) curves for PbS QD-TBAI (860 nm), PbS QD-EDT (1595 nm), and 860 nm PbS QD-TBAI/1595 nm PbS QD-EDT homojunction films.

**Figure S16.** AFM topographical images and corresponding height profiles for 2L, 4L, and 6L devices, confirming a consistent total thickness of 93±8 nm.

**Figure S17.** Simulated band diagrams for a unit structure of the cascade junction, i.e., PbS QD-TBAI/PbS QD-EDT/PbS QD-TBAI, with different single-layer thickness (t). The resulting *Φ_pn_* is shown for: (a) t=100 nm, *Φ_pn_*=0.21 eV; (b) t=200 nm, *Φ_pn_*=0.28 eV; and (c) t=300 nm, *Φ_pn_*=0.38 eV.

**Figure S18.** Simulated PSC of the 2L device with different single layer thickness of 100, 150, and 200 nm.

**Figure S19.** Performance of the champion device with 4L cascade-junction structures. (a) Rise time, (b) fall time, and (c) I-V characteristics and calculated R and EQE at V_bias_=-0.1 V under 1535 nm continuous laser illumination when operating as a photodetector; (d) PSC under pulsed 808 nm laser illumination and calculated PPF and retention time when operating as a synapse at V_bias_=0.2 V. (e) Linear dynamic range measured at -0.1 V with intensity ranging from 100 μW/cm^2^ to 500 mW/cm^2^.

**Figure S20.** Long-term stability of the champion device stored under ambient conditions. (a)–(e) I-V characteristics of the device measured every 24 hours from the as-fabricated state (day 0) to day 4. (f) Corresponding EQE degradation as a function of time.

**Figure S21.** (a) Schematic illustration of the denoising mechanism with the PbS QD optoelectronic synapses. Red pixels denote the useful signal, and blue pixels represent noise. The PSC of the synapse device pixel under these two signal associated illumination conditions. (b) Illustrative examples of original (left), 50% noise introduced (middle), and denoised images of handwritten digits. (c) Recognition accuracy of the original image, the same image with a 50 % noise intensity, and the same image after the denoising process.

**Image Denoising and Digit Recognition Tasks**

A simulated image denoising and digit recognition task using the MNIST dataset is performed. As illustrated in **Figure S13a**, to map the information of an image of digits onto a PbS QD optoelectronic synapse array, the local brightness of these 28 × 28 pixels is encoded into the duration of light pulses that are illuminated on the corresponding synapse device. The synapse array acts as a hardware-level high-pass filter by leveraging its intrinsic PSC plasticity. Specifically, high-intensity signals from the digit features trigger a persistent PSC (red curve), whereas low-intensity noise signals are unable to generate accumulative PSC but rapidly decay to zero (blue curve). This behavior mimics the signal-filtering mechanism observed in the human peripheral visual system, where redundant or weak stimuli are filtered out before data transmission to the visual cortex.

Here, Gaussian noise (mean 0.5, variance 0.1) is added to 50% of the original image pixels, making the digits less distinguishable. However, after applying the denoising process through the synapse array described above, clear digit features restore (**Figure S13b)**. To quantitatively assess the enhancement in data quality, a three-layer artificial neural network (ANN) is employed for pattern classification. As shown in **Figure S13c**, the ANN trained with processed images achieves a peak accuracy of over 96%, which closely follows the performance of the noise-free baseline (~98%), while the group with noisy background only reaches an accuracy ~30% lower. These results highlight the capability of the PbS QD optoelectronic synapses to perform energy-efficient, front-end signal processing.

**Figure S22.** Optical photos of the experimental setup for stages I through IV, corresponding to Figure 4(e).

Two independent 1550 nm light sources are arranged in the testing setup. The experimental demonstration proceeds through four stages: (i) Stage I: Both IR light sources remain turned off to establish the initial dark baseline. (ii) Stage II: Light Source 1 is switched on, serving as a continuous, constant illumination (5 mW/cm²) to simulate a static ambient background. (iii) Stage III: Light Source 2 (5 mW/cm²) is turned on and translated from a distance toward the sample, briefly irradiating the device before moving away, thereby simulating the transient passage of a dynamic target. (iv) Stage IV: Light Source 2 is moved away from the sample, while Light Source 1 remains as the static background.

**Figure S23.** Simulated PSC response at low bias and intrinsic static background suppression at 0.06 V bias under stepped light intensities: 5 mW/cm^2^ (baseline, 0-5 s), 10 mW/cm^2^ (target stimulus, 5-25 s), and a return to 5 mW/cm^2^ (25-35 s). (f) Simulated temporal SNR comparison under stepped light intensities for synapse mode II (0.5 V), Synapse mode I (0.06 V) and photodetector mode (-0.5 V).

**Figure S24.** The device model for the COMSOL simulation studies.

**Table S2. Comparison of the photodetection performance and synaptic functionality of this work with previously reported dual-mode, QD based photodetectors and SWIR synapses**

| **Sample** | **Wavelength (nm)** | | **Responsivity (mA/W)** | | **Response time (μs)** | | **Synapse mode (Y/N)** | | **Background suppression (Y/N)** | **Reference** |
| --- | --- | --- | --- | --- | --- | --- | --- | --- | --- | --- |
| CoO_x_/InGaN NW | 450 | | 88.6 | | 75.2/45.9 | | Y | N | | ^1^ |
| MoTe_2_/MoS_2_ | 532 | | 300 | | 188/45 | | Y | N | | ^2^ |
| p-GaN/n-AlGaN/n-GaN | 265 | | 10.5 | | NA/NA | | Y | N | | ^3^ |
| GaN/Ga_2_O_3_/GaN | 254 | | 4.22 | | 118/167 | | Y | N | | ^4^ |
| Au/PbS/Al_2_O_3_/Si/InGa | 970 | | 543.6 | | 670/6760 | | Y | N | | ^5^ |
| 5L Multijunction Cascade PbS QD | 1550 | | 1.9×10^4^ | | 9/8.1 | | N | N | | ^6^ |
| ITO/ZnO/PbS QD/Au | 1550 | | 1000 | | NA/0.01 | | N | N | | ^7^ |
| TeSeO/InZnO | 1550 | / | | / | | N | | | N | ^8^ |
| PbS QDs/P3HT/Si NM | 1342 | / | | / | | N | | | N | ^9^ |
| PdSe2/pentacene | 3000-4250 | / | | / | | N | | | N | ^10^ |
| MoS_2_/Ta_2_NiS_5_ | 1550 | 1.2×10^-5^ | | 6.37×10^4^/  5.86×10^4^ | | Y | | | N | ^11^ |
| Multijunction PbS QD | 1535 | | 430 | | 2.2/2.1 | | Y | Y | | This Work |

**Table S3. Parameters used in COMSOL Multiphysics simulations**

|  | **PbS QD-TBAI** | **PbS QD-EDT** |
| --- | --- | --- |
| Thickness (nm) | 200 | 300 |
| Bandgap (eV) | 1.3 | 1.3 |
| Electron affinity (eV) | 4.04 | 3.85 |
| Permittivity | 14.5 | 14.5 |
| CB effective density of states (1/cm^3^) | 1.0076×10^18^ | 1.0076×10^18^ |
| VB effective density of states (1/cm^3^) | 8.84×10^17^ | 8.84×10^17^ |
| Electron mobility (cm^2^/Vs) | 1×10^-7^ | 1×10^-7^ |
| Hole mobility (cm^2^/Vs) | 1×10^-7^ | 1×10^-7^ |
| N_donor_ (1/cm^3^) | 2.7×10^8^ | 0 |
| N_acceptor_ (1/cm^3^) | 0 | 2.7×10^15^ |
| Refractive index (imaginary part) | 0.1 | 0.1 |

**References**

1.	Chen, W. *et al.* Bioinspired Reconfigurable Vision Sensor Based on InGaN Semiconductor/Hydrogel Heterostructure for Dynamic-Static Fusion Perception. *Advanced Materials* **n/a**, e12894.

2.	Ouyang, Y. *et al.* Gate-Tunable Dual-Mode Optoelectronic Device for Self-Powered Photodetector and Optoelectronic Synapse. *Advanced Science* **12**, 2416259 (2025).

3.	Luo, Y. *et al.* A single diode with integrated photosensing, memory and processing for neuromorphic image sensors. *Nat Electron* **9**, 404–413 (2026).

4.	Feng, S. *et al.* Dual-Mode Conversion of Photodetector and Neuromorphic Vision Sensor via Bias Voltage Regulation on a Single Device. *Advanced Materials* **35**, 2308090 (2023).

5.	Fu, C. *et al.* Dual-Mode Semiconductor Device Enabling Optoelectronic Detection and Neuromorphic Processing with Extended Spectral Responsivity. *Advanced Materials* **36**, 2409406 (2024).

6.	Zhou, W. *et al.* Quantum-Tuned Cascade Multijunction Infrared Photodetector. *ACS Nano* **17**, 18864–18872 (2023).

7.	Vafaie, M. *et al.* Colloidal quantum dot photodetectors with 10-ns response time and 80% quantum efficiency at 1,550 nm. *Matter* **4**, 1042–1053 (2021).

8.	Zhu, L. *et al.* Bioinspired TeSeO/InZnO Optoelectronic Synapse for Broadband UV–Visible–NIR Sensing and Multifunctional Reservoir Computing. *Nano Lett.* **26**, 1499–1507 (2026).

9.	Wang, Y. *et al.* Silicon-Nanomembrane-Based Broadband Synaptic Phototransistors for Neuromorphic Vision. *Nano Lett.* **23**, 8460–8467 (2023).

10.	Gao, H. *et al.* Bio-inspired mid-infrared neuromorphic transistors for dynamic trajectory perception using PdSe2/pentacene heterostructure. *Nat Commun* **16**, 5241 (2025).

11.	Ling, S. & Hou, P. Self-powered infrared detection, polarization sensing, and visual synaptic behavior in a multifunctional MoS2/Ta2NiS5 heterojunction. *J. Mater. Chem. C* **12**, 17206–17214 (2024).
